# Programmed death-ligand 1 (PD-L1) expression in tumour cell and tumour infiltrating lymphocytes of HER2-positive breast cancer and its prognostic value

**DOI:** 10.1038/s41598-017-11905-7

**Published:** 2017-09-15

**Authors:** Ahrong Kim, So Jeong Lee, Young Keum Kim, Won Young Park, Do Youn Park, Jee Yeon Kim, Chang Hun Lee, Gyungyub Gong, Gi Yeong Huh, Kyung Un Choi

**Affiliations:** 1 Department of Pathology, BioMedical Research Institute, Pusan National University Hospital, Pusan, Korea; 2 Department of Pathology, Pusan National University Yangsan Hospital, Yangsan, Korea; 3 Department of Pathology, Asan Medical Center, University of Ulsan College of Medicine, Seoul, Korea

## Abstract

Immunotherapy targeting PD-1/PD-L1 axis showed benefits in cancer. Prognostic significance of tumour infiltrating lymphocytes (TILs) has been determined. We evaluated PD-L1 protein expression in tumour cells and TILs, PD-L1 mRNA level and various histopathologic factors including TILs using 167 formalin-fixed paraffin embedded tissues and 39 fresh tissue of HER2-positive breast cancer. TILs level and PD-L1 expression in tumour cells and TILs were significantly correlated one another. PD-L1 positivity in tumour cells was associated with high histologic grade and high TILs level (p < 0.001, both). High PD-L1 immunoscore in TILs and high total immunoscore (in tumour cells and TILs) of PD-L1 were correlated with high histologic grade (p = 0.001 and p < 0.001, respectively), absence of lymphovascular invasion (p = 0.012 and p = 0.007, respectively), negative hormone receptor expression (p = 0.044 and p = 0.001, respectively) and high TILs level (p < 0.001, both). High PD-L1 mRNA expression was associated with high TILs level (p < 0.001, both). PD-L1 positivity in tumour cells was associated with better disease-free survival in HR−/HER2+ breast cancer (p = 0.039). PD-L1 expression in tumour cells and TILs are significantly associated with TILs level in HER2-positive breast cancer. PD-L1 expression in tumour cells might be positive prognostic factor in HR−/HER2+ breast cancers.

## Introduction

Recent progress in the field of immunology supports the theory that the immune system has a role in cancer development through “cancer immunoediting”. This encompasses three processes: elimination, equilibrium and escape^1^. Evading immune destruction was added as a component of the hallmarks of cancer by Hanahan and Weinberg in 2011^2^, with emphasis on the tumour microenvironment. There are three mechanisms of immune escape in cancer, including loss of both antigenicity and immunogenicity, and an establishment of an immunosuppressive microenvironment^3^. To lose immunogenicity, tumour cells use immune checkpoints, which are essential for the maintenance of self-tolerance under normal physiological conditions. Immune checkpoints, including co-stimulatory and inhibitory signals, regulate the level of the T cell response. Inhibitory ligands and receptors, which regulate T cell effector functions, are generally overexpressed in tumour or non-transformed cells in the tumour microenvironment^4^.

Immune checkpoint blocking agents targeting the cytotoxic T lymphocyte-associated antigen 4 (CTLA4) and programmed death -1 (PD-1)/programmed death-ligand 1 (PD-L1) axis have been developed and possess notable clinical efficacy^5–13^. Remarkably, Schadendorf *et al*.^5^ observed a plateau in the survival curves in a meta-analysis of overall survival involving the use of anti-CTLA4 (ipilimumab) in patients with advanced melanoma, which started at approximately three years. This suggests that immunotherapy could be a potentially curative treatment option for advanced malignancies. Despite the evidences surrounding the clinical benefits of immune checkpoint blocking agents, particularly those targeting the PD-1/PD-L1 axis, the value of PD-L1 expression as a predictive biomarker for patient response to anti-PD-1/PD-L1 therapy has not been established. Although the expression of PD-L1 in tumour cells was considered a potential biomarker for PD-1/PD-L1 blocking agents, it has demonstrated effectiveness even in PD-L1 negative patients^14, 15^. Furthermore, several reports have demonstrated that lymphocyte PD-L1 expression was associated with the response to anti-PD-1/PD-L1 therapy^10–12^. Moreover, the prognostic significance of PD-L1 expression has not been conclusively determined.

The prognostic impact of tumour infiltrating lymphocytes (TILs) has been reported in various organs, including the breast, and specifically in human epidermal growth factor receptor 2 (HER2)-positive and triple-negative breast cancers (TNBCs)^16^. In HER2-positive breast cancers, treatment with trastuzumab appears to boost the immune system response^17^. A high level of TILs is associated with a better response to trastuzumab, and is a good prognostic factor in HER2-positive breast cancers^18, 19^. Moreover, there is a consensus regarding the methodology for evaluating histological levels of TILs in breast cancers^20^.

In addition to the progress made in immunotherapy and cancer immunology, Teng *et al*.^21^ classified cancers based on TIL and PD-L1 expression and proposed immunotherapeutic strategies according to their classification. As previously mentioned, immune mechanisms are associated with the efficacy of trastuzumab treatment, and several attempts have been made to use immune checkpoint inhibitors in combination with trastuzumab. Ado-trastuzumab emtansine (T-DM1) is an antibody-drug conjugate consisting of the monoclonal antibody trastuzumab linked to the cytotoxic agent emtansine. The combination of T-DM1 and anti-CTLA4 or anti-PD-1 antibodies, elicited responses in tumour xenografts that had previously been resistant to T-DM1 monotherapy in a mouse model^22^. An international phase Ib/II trial (ClinicalTrial.Gov Identifier: NCT02129556) involving trastuzumab in combination with the anti-PD-1 antibody is currently being investigated. In a mouse model, the synergistic effect of anti-PD-1 agent on trastuzumab therapy was demonstrated in HER2-positive breast cancer^17^. However, the immune checkpoints in HER2-positive breast cancers, particularly PD-L1, are not completely elucidated. Whilst reports indicate the significance of PD-L1 expression in TILs for predicting the response to anti-PD-1/PD-L1 inhibitors, many recent studies have focused on the expression of PD-L1 in tumour cells alone. Despite efforts to establish a combination therapy involving trastuzumab and anti-PD-1/PD-L1 inhibitors, PD-L1 expression in HER2-positive breast cancers has not been fully determined.

In this study, the expression of PD-L1 in tumour cells and TILs and the relationship between the expression levels of PD-L1 and various clinicopathological parameters, including TILs, were evaluated. The prognostic significance of PD-L1 expression was also assessed.

## Results

### Clinicopathological characteristics of HER2-positive breast cancers

A cohort of 167 patients with HER2-positive breast cancers was included in our study. Patient age ranged from 23 to 85 years (mean: 52.59 ± 10.126 years) and the size of the breast tumours ranged from 0.2 to 15.5 cm (mean 3.213 ± 1.773 cm). There were 93 (55.7%) and 74 cases (44.3%) of left and right breast cancers, respectively. Eighty-nine cases (53.3%) were of hormone receptor (HR)+/HER2+ breast cancers and 78 cases (46.7%) were of HR−/HER2+ breast cancers. Sixty-one patients (36.5%) had TIL levels of 10% or less, 74 patients (44.3%) had TIL levels between 20% and 60%, and the remaining 32 patients (19.2%) had TIL levels of more than 60%. One hundred and two cases (61.1%) were categorized as having a low level of tertiary lymphoid structures (TLSs) around the ductal carcinoma *in situ* (DCIS), the remaining 65 cases (38.9%) were categorized as having a high level of TLSs around the DCIS. Patients were also grouped by abundance of TLSs around invasive margins, 109 patients (65.3%) and the remaining 58 patients (34.7%) were classified as having a high and low level of TLSs respectively (Supplementary Table 1).

### Protein expression levels of PD-L1 and TILs in HER2-positive breast cancers

**Figure 1:**
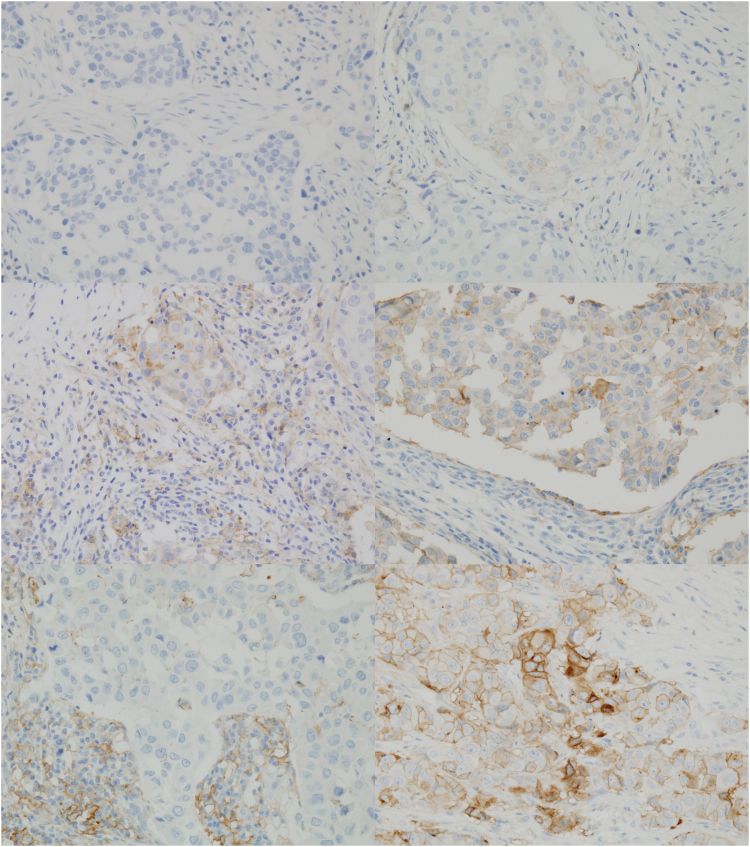
PD-L1 expression by immunohistochemistry (**A**) Negative for PD-L1 expression in tumour cells and TILs (**B**) PD-L1 expression in tumour cells (arrow head) with intensity of 1 (**C**) PD-L1 expression in both TILs (arrow) and tumour cells with intensity of 2 (**D**) PD-L1 expression in tumour cells with intensity of 2 (**E**) PD-L1 expression in TILs with intensity of 3 (**F**) PD-L1 expression in tumour cells with intensity of 3.

Eighty-one patients (48.5%) showed positive immunostaining for PD-L1 in tumour cells according to the Allred scoring system (Fig. 1). High levels of PD-L1 expression in TILs were identified in 51 cases (30.5%) (Fig. 1). Fifty-three cases (31.7%) were categorized by their total immunoscore as high level for tumour cells and TILs. According to Teng’s classification^21, 23^, 28 cases (16.8%) were type I tumours, 82 cases (49.1%) were type II, 53 cases (31.7%) were type III and four cases (2.4%) were type IV (Supplementary Table 2). Among 81 cases of positive immunostaining for PD-L1 in tumour cells, 43 cases (53.1%) showed high expression level of PD-L1 in TILs. Moreover, among the 86 cases of negative PD-L1 immunostaining in tumour cells, 8 cases (9.3%) showed high PD-L1 expression in TILs (Supplementary Table 3).

The results of immunohistochemical staining were compared to that of the corresponding western blot analysis performed using fresh tissue, and the results showed a relatively strong association (Supplementary Fig. 1). In correlation analyses, TIL level, the immunoscore for PD-L1 in tumour cells, and the immunoscore for PD-L1 in TILs showed a significant positive association with each other (Table 1).

**Table 1:** Correlation between the TILs level, PD-L1 immunoscore in tumour cells, PD-L1 immunoscore in TILs and total PD-L1 immunoscore in tumour cells and TILs among 167 HER2-positive breast cancers*.

	Immunoscore of PD-L1 in tumour cells	Immunoscore of PD-L1 in TILs	Total immunoscore of PD-L1 in tumour cells and TILs
TILs level	0.549** (*p* < 0.001)	0.648 (*p* < 0.001)	0.666(*p* < 0.001)
Immunoscore of PD-L1 in tumour cells		0.604 (*p* < 0.001)	0.813 (*p* < 0.001)
Immunoscore of PD-L1 in TILs			0.937 (*p* < 0.001)

*Spearman correlation analysis was performed. **Spearman correlation coefficient.

The association between PD-L1 protein expression and variable clinicopathological factors is shown in Table 2. Positive immunostaining for PD-L1 in tumour cells was significantly associated with various histopathological factors, including high histologic grade (*p* < 0.001), high TIL level (*p* < 0.001), and an abundance of TLSs around the invasive component (*p* = 0.002). High PD-L1 immunoscores for TILs was significantly correlated with high histologic grade (*p* = 0.001), absence of lymphovascular invasion (*p* = 0.012), absence of HR expression (*p* = 0.044), high levels of TILs (*p* < 0.001) and abundance of TLSs around the invasive component (*p* = 0.034). High levels of total PD-L1 immunoscore (in tumour cells and TILs) showed significant association with high histologic grade (*p* < 0.001), absence of lymphovascular invasion (*p* = 0.007), absence of HR expression (*p* = 0.001), and high levels of TILs (*p* < 0.001).

**Table 2:** Correlation between PD-L1 protein expression by immunohistochemistry and variable clinicopathologic parameters in 167 HER2-positive breast cancers

	PD-L1 expression on tumour cells by Allred scoring system		*p*-value	PD-L1 expression on TILs		*p*-value	Total PD-L1 expression on tumour cells and TILs		*p*-value
	Negative (Number (%))*	Positive (Number (%))*		Low (Number (%))*	High (Number (%))*		Low (Number (%))*	High (Number (%))*	
Age (years), mean ± SD**	52.94 ± 10.26	52.21 ± 10.02	0.642	52.84 ± 10.76	52.00 ± 8.58	0.621	52.49 ± 10.73	52.79 ± 8.77	0.859
Size (cm), mean ± SD**	3.130 ± 2.047	3.300 ± 1.434	0.538	3.187 ± 1.626	3.271 ± 2.086	0.590	3.27 ± 2.010	3.07 ± 1.100	0.848
Side			0.353			0.616			0.504
Right	51 (59.3)	42 (51.9)		63 (54.3)	30 (58.8)		61 (53.5)	32 (60.4)	
Left	35 (40.7)	39 (48.1)		53 (45.7)	21 (41.2)		53 (46.5)	21 (39.6)	
Histologic grade			<0.001			0.001			<0.001
Grade 1, 2	43 (50.0)	19 (23.5)		53 (45.7)	9 (17.6)		54 (47.4)	8 (15.1)	
Grade 3	43 (50.0)	62 (76.5)		63 (54.3)	42 (82.4)		60 (52.6)	45 (84.9)	
Nuclear grade			0.227			0.120			0.192
Grade 1, 2	18 (20.9)	11 (13.6)		24 (20.7)	5 (9.8)		23 (20.2)	6 (11.3)	
Grade 3	68 (79.1)	70 (86.4)		92 (79.3)	46 (90.2)		91 (79.8)	47 (88.7)	
Necrosis			0.256			1.000			0.489
Absent	33 (38.4)	24 (29.6)		40 (34.5)	17 (33.3)		41 (36.0)	16 (30.2)	
Present	53 (61.6)	57 (70.4)		76 (65.5)	34 (66.7)		73 (64.0)	3 (69.8)	
Lymphovascular invasion			0.062			0.012			0.007
Absent	40 (46.5)	50 (61.7)		55 (47.4)	35 (68.6)		53 (46.5)	37 (69.8)	
Present	46 (53.5)	31 (38.3)		61 (52.6)	16 (31.4)		61 (53.5)	16 (30.2)	
Lymph node metastasis			0.758			0.503			0.507
Absent	39 (45.3)	39 (48.1)		52 (44.8)	26 (51.0)		51 (44.7)	27 (50.9)	
Present	47 (54.7)	42 (51.9)		64 (55.2)	25 (49.0)		63 (55.3)	26 (49.1)	
Stage			0.722			0.340			0.256
Early (stage I, II)	66 (76.7)	60 (74.1)		85 (73.3)	41 (80.4)		83 (72.8)	43 (81.1)	
Advanced (stage III, IV)	20 (23.3)	21 (25.9)		31 (26.7)	10 (19.6)		31 (27.2)	10 (18.9)	
HR expression			0.217			0.044			0.001
Positive	50 (58.1)	39 (48.1)		68 (58.6)	21 (41.2)		71 (62.3)	18 (34.0)	
Negative	36 (41.9)	42 (51.9)		48 (41.4)	30 (58.8)		43 (37.7)	35 (66.0)	
TILs level***			<0.001			<0.001			<0.001
≤10%	46 (53.5)	15 (18.5)		57 (49.1)	4 (7.8)		58 (50.9)	3 (5.7)	
20–60%	36 (41.9)	38 (46.9)		49 (42.2)	25 (49.0)		47 (41.2)	27 (50.9)	
>60%	4 (4.7)	28 (34.6)		10 (8.6)	22 (43.1)		9 (7.9)	23 (43.4)	
TLSs around DCIS			0.112			0.493			0.307
Low	58 (67.4)	44 (54.3)		73 (62.9)	29 (56.9)		73 (64.0)	29 (54.7)	
High	28 (32.6)	37 (45.7)		43 (37.1)	22 (43.1)		41 (36.0)	24 (45.3)	
TLSs around invasion			0.002			0.034			0.057
Low	66 (76.7)	43 (53.1)		82 (70.7)	27 (52.9)		80 (70.2)	29 (54.7)	
High	20 (23.3)	38 (46.9)		34 (29.3)	24 (47.1)		34 (29.8)	24 (45.3)	

*except for age and sizes. **The t-test was performed and the equality of variances was assumed. SD, standard deviation. ***The linear-by-linear association was carried out. The Pearson’s Chi-square test or Fisher’s exact test was used as appropriate for the other variable.

### mRNA expression of PD-L1 in HER2-positive breast cancers

The fold difference value of PD-L1 mRNA ranged from 0.0231 to 1.1464 (mean: 0.3729 ± 0.0422).Twenty-three cases (59.0%) and the remaining 16 cases (41.0%), were categorized as having low and high expression levels of PD-L1 mRNA respectively based on the mean fold difference value. In the t-test, the fold difference value of the PD-L1 mRNA showed difference between the low and high total immunoscore group (0.3153 ± 0.0495 vs 0.4757 ± 0.7195, respectively, *p* = 0.068), though it was not statistically significant. In correlation analyses, the TIL level, each PD-L1 immunoscore for tumour cells and TILs, the total PD-L1 immunoscore in tumour cells and TILs, and mRNA expression levels of PD-L1 showed a significant positive association with one another (Table 3). High levels of PD-L1 mRNA expression were significantly associated with high TIL level (*p* = 0.001) and there was no significant association between mRNA levels of PD-L1 and other clinicopathological factors (Table 4).

**Table 3:** Correlation between the mRNA level of PD-L1, TILs level, PD-L1 immunoscore in tumour cell, PD-L1 immunoscore in TILs and total PD-L1 immunoscore in tumour cells and TILs among 39 patients with fresh tissue*

	Immunoscore of PD-L1 on tumour cells	Immunoscore of PD-L1 on TILs	Total immunoscore of PD-L1 on both tumour cells and TILs	mRNA level of PD-L1
TILs level	0.494** (*p* = 0.001)	0.648 (*p* < 0.001)	0.662 (*p* < 0.001)	0.532 (*p* < 0.001)
Immunoscore of PD-L1 on tumour cells		0.500 (*p* = 0.001)	0.764 (*p* < 0.001)	0.404 (*p* = 0.011)
Immunoscore of PD-L1 on TILs			0.917 (*p* < 0.001)	0.348 (*p* = 0.030)
Total immunoscore of PD-L1 on tumour cells and TILs				0.469 (*p* = 0.003)

*Spearman correlation analysis was performed. **Spearman correlation coefficient.

**Table 4:** Correlation between mRNA level of PD-L1 and variable clinicopathologic parameters in 39 HER2-positive breast cancers with fresh tissue.

	mRNA level of PD-L1		*p*-value
	Low (number (%))*	High (number (%))*	
Age, mean ± SD **	55.65 ± 10.14 years	55.00 ± 10.85 years	0.849
Size, mean ± SD **	3.98 ± 2.09 cm	4.57 ± 3.16 cm	0.488
Side			0.571
Right	15 (65.2)	9 (56.3)	
Left	8 (34.8)	7 (43.8)	
Histologic grade			1.000
Grade 1, 2	8 (34.8)	6 (37.5)	
Grade 3	15 (65.2)	10 (62.5)	
Nuclear grade			0.432
Grade 1, 2	6 (26.1)	2 (12.5)	
Grade 3	17 (73.9)	14 (87.5)	
Necrosis			1.000
Absent	6 (26.1)	4 (25.0)	
Present	17 (73.9)	12 (75.0)	
Lymphovascular invasion			0.743
Absent	11 (47.8)	6 (37.5)	
Present	12 (52.2)	10 (62.5)	
Lymph node metastasis			0.291
Absent	9 (39.1)	3 (18.8)	
Present	14 (60.9)	13 (81.3)	
Stage			0.112
Early (stage I, II)	15 (65.2)	6 (37.5)	
Advanced (stage III, IV)	8 (34.8)	10 (62.5)	
HR expression			0.192
Positive	13 (56.5)	5 (31.3)	
Negative	10 (43.5)	11 (68.8)	
TILs level***			0.001
≤10%	11 (47.8)	1 (6.3)	
20–60%	12 (52.2)	10 (62.5)	
60%	0 (0.0)	5 (31.3)	
TLSs around DCIS			0.264
Low	19 (82.6)	10 (62.5)	
High	4 (17.4)	6 (37.5)	
TLSs around invasive component			0.471
Low	18 (78.3)	10 (62.5)	
High	5 (21.7)	6 (37.5)	

*except for age and sizes. **The t-test was performed and the equality of variances was assumed. SD, standard deviation. ***The linear-by-linear association was carried out. The Pearson’s Chi-square test or Fisher’s exact test was used as appropriate for the other variables.

### Prognostic significance of PD-L1 expression in HER2-positive breast cancers

The median follow-up time was 1157 days (43–1748). During the follow-up period, there were five cases of local recurrence (2.99%) and 14 cases of distant metastasis (8.38%). Five patients died because of the disease (3.0%).

**Figure 2:**
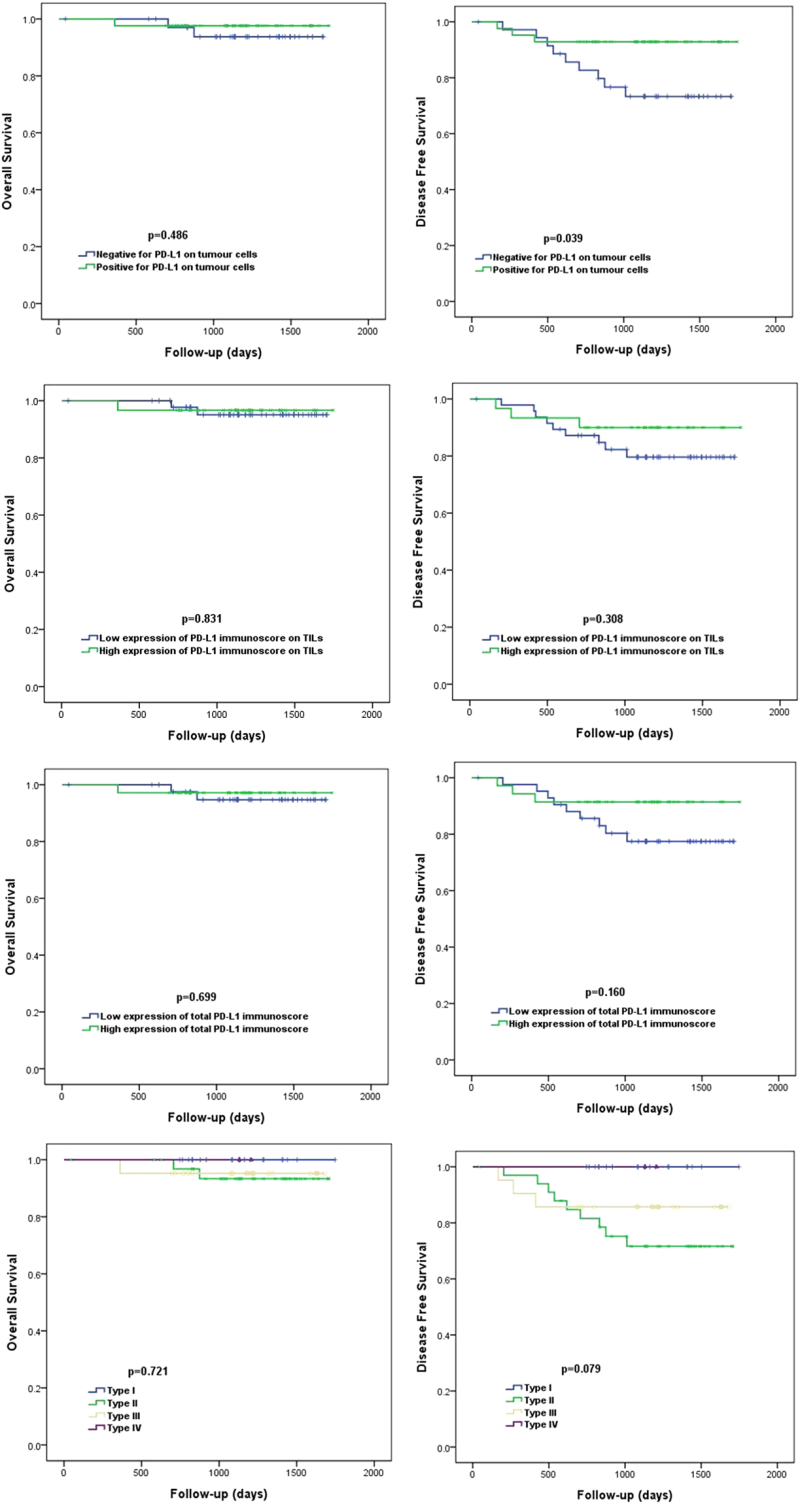
Overall survival and disease free survival in HR−/HER2+ breast cancers (**A**) Overall survival according to PD-L1 expression in tumour cells (**B**) Disease-free survival according to PD-L1 expression in tumour cells (**C**) Overall survival according to level of PD-L1 immunoscore in TILs (**D**) Disease-free survival according to level of PD-L1 immunoscore in TILs (**E**) Overall survival according to level of total PD-L1 immunoscore (**F**) Disease free survival according to level of total PD-L1 immunoscore (**G**) Overall survival according to tumour types (**H**) Disease free survival according to tumour types.

For survival analysis, the TIL level was dichotomized and the patients were grouped into four categories according to Teng’s classification^21, 23^. The expression of PD-L1 was not significant for the prognosis of total HER2-positive breast cancers and HR+/HER2+ breast cancers (Supplementary Figs 2 and 3). PD-L1 positivity in tumour cells was associated with a better disease-free survival rate in patients with HR−/HER2+ breast cancer (*p* = 0.039, Fig. 2). However, PD-L1 positivity in tumour cells was not associated with disease-free survival on multivariate analysis (Supplementary Table 4). The PD-L1 immunoscore of TILs and the total immunoscores of tumour cells and TILs were not prognostic for HR−/HER2+ breast cancers (Fig. 2). Type II tumours (tumours negative for PD-L1 with low TIL level) were associated with poor disease-free survival in HR−/HER2+ breast cancers, however this was not statistically significant (*p* = 0.079, Fig. 2).

## Discussion

PD-L1 (also known as CD274 and B7-H1) is a ligand of PD-1. PD-1 is a transmembrane receptor and an important negative immune controller, regulating the activation, exhaustion, and tolerance of T cells and the resolution of inflammation. The PD-1 pathway is activated upon engagement of its ligands, PD-L1 or PD-L2, and can modulate T cell activity in several ways. The interaction of PD-1 with its ligand induces epigenetic modification in T cells, which alters transcription factor expression and reduces the phosphorylation of T cell receptor (TCR) signalling molecules. Additionally, the PD-1 signal stimulates the expression of proteins that decrease T cell proliferation and cytokine production, thus decreasing T cell survival and killing capacity^24^. PD-L1 is expressed on hematopoietic cells and non-hematopoietic cells such as epithelial, vascular endothelial, tumour and stromal cells.

Tumour cells use two mechanism for expressing PD-L1: adaptive immune resistance (tumour extrinsic mechanism) and innate immune resistance (tumour intrinsic mechanism)^4^. The adaptive immune resistance mechanism of PD-L1 upregulation is an adaptation to an endogenous tumour-specific immune response. The tumour cells use the normal physiology that occurs during pathogen infection to protect the host from immune-mediated damage. Simply, PD-L1 is induced in tumour cells in response to inflammatory signals such as interferons (IFNs). In a previous study of HER2-positive breast cancers, expression of Myxovirus resistance A (MxA), an excellent marker of IFNs activity, in tumour cells was significantly associated with high TIL levels^19^. Furthermore, the current study found that TIL level, PD-L1 immunoscore in tumour cells and in TILs, and the total PD-L1 immunoscore in tumour cells and TILs, significantly correlated with each other. Innate immune resistance demonstrates that PD-L1 is driven by constitutive oncogenic signalling pathways in the tumour cells. Constitutive anaplastic lymphoma kinase (ALK) signalling has been reported to induce PD-L1 expression^25^. Phosphatase and tensin homolog (PTEN) loss or downregulation, which reflects the phosphatidulinositol-4,5-biphosphate 3-kinase/protein kinase B (PI3K/Akt) pathway, was related to PD-L1 expression in TNBCs and glioblastoma^26, 27^. The current study showed that 53 cases (31.7%) involved type III tumours (positive for PD-L1 with low TIL levels) suggesting tumour intrinsic induction. Tokunaga *et al*.^28^ demonstrated that Akt activation was significantly associated with HER2 overexpression in breast cancers. However, the intrinsic induction mechanism of PD-L1 in HER2-positive breast cancer should be determined.

The expression levels of PD-L1 have been reported with a diverse range, and many studies have focused on the expression of PD-L1 in tumour cells alone^29–35^. This study showed that 81 patients (48.5%) were positive for PD-L1 in tumour cells, whereas 51 cases (30.5%) showed high levels of PD-L1 expression in TILs. Overall, 89 patients (53.3%, Supplementary Table 3), with either PD-L1 positivity in tumour cells or high level of PD-L1 in TILs, could be considered as potential responders to anti-PD-1/PD-L1 blocking agents after considering evidence from previous clinical trials in other malignancies^10–12^.

Results regarding the prognostic significance of PD-L1 in breast cancers are conflicting. Several reports suggest a good prognostic role of PD-L1 in breast cancer^29–32, 36^, although certain investigators have demonstrated an association between PD-L1 expression and poor prognosis^33–35^. Our study revealed that PD-L1 expression in tumour cells was significantly associated with a better disease-free survival rate in patients with HR−/HER2+ breast cancers. This positive prognostic effect could be an unexpected result, considering that PD-1/PD-L1 axis plays an important role in the immune escape of cancer. Unlike CTLA4, which is induced in the initial T cell activation stage, PD-L1 is induced on tumour cells in response to inflammatory signals such as IFNs, as described above with respect to adaptive immune resistance^4^. This implies that the antitumor immune microenvironment precedes the induction of PD-L1 in tumour cells. In other words, TILs in tumour microenvironment still affect the survival benefits despite partial disturbance of anti-tumour effects of TILs by PD-L1 expression^29^. Interestingly, type I and type IV tumours showed improved disease-free survival; however, type II tumours (PD-L1 negative tumours with low TIL level) were associated with poor disease-free survival, and type III tumours had intermediate disease-free survival rates in HR−/HER2+ breast cancers, although it was not statistically significant. Better disease-free survival of type III tumours than type II tumours in HR−/HER2+ breast cancers suggests probable positive prognostic role of PD-L1 expression regardless of the status of the tumour microenvironment. Further *in vitro* studies are required to clarify the prognostic value of PD-L1 in cancer. Type II tumours can be described as tumours with immunological ignorance^21^. For such tumours, adoptive T cell therapy with the delivery of costimulatory signals, the blocking of inhibitory signals and vaccination could be considered. Adoptive T cell therapy is a form of immunotherapy whereby *ex vivo* isolation and expansion of antigen-specific T cells is performed and they are transferred back into patients that already showed clinical efficacy in recurrent viral infections^37^. Furthermore, Bald *et al*. showed that reversion of immune ignorance with peritumoural injections of the immunostimulant, polyinosinic:polycytidylic acid (poly:IC), induced upregulation of PD-L1 expression in a mouse model of human immune cell-poor melanoma. Additionally, they demonstrated the synergistic effect of anti-PD-1 therapy with immunostimulants^38^. In the present study, type III tumours showed intermediate outcomes in disease-free survival compared with the remaining tumour types in patients with HR−/HER2+ breast cancers. As mentioned above, intrinsic tumour expression of PD-L1 is associated with constitutive oncogenic signalling. However, these results suggest that blocking anti-PD-1/PD-L1 is unlikely to induce T cell response to the tumour because the tumour microenvironment does not have enough TILs to elicit effective anti-tumour immunity. An approach similar to that used for type II tumours involving the recruitment of TILs into the tumour microenvironment can also be used for this group of patients.

IFNs, inducers of PD-L1, stimulate the expression of human leukocyte antigen (HLA)^39, 40^. Moreover, Lee *et al*. recently demonstrated that HLA expression was negatively associated with estrogen receptor expression and positively correlated with high TILs level and IFN-associated molecules in breast cancer^41^. However, the biologic determinants of PD-L1 expression in tumour cells as a positive prognostic factor in HR negative tumours, but not in HR positive tumours, should be further explored.

Studies with large cohorts with long term follow up periods are required to classify all consecutive breast cancers, as each type should have a different treatment strategy. Furthermore, functional studies are required to understand the intrinsic tumour expression of PD-L1 in breast cancer. The importance of defining the molecular pathways that regulate the expression of immune checkpoints cannot be overemphasized, as immune checkpoint intervention will likely lead to clinical benefits.

In conclusion, TIL levels and PD-L1 protein expression in tumour cells and in TILs are significantly correlated with one another. High expression of PD-L1 mRNA was significantly associated with high TIL levels. Protein expression of PD-L1 in tumour cells was significantly associated with a better disease-free survival rate in HR−/HER2+ breast cancers.

## Materials and Methods

### Patients and tissue specimens

A total of 167 patients with HER2-positive breast cancer were included in our study. Patients were recruited following surgery for primary breast cancer between 2011 and 2013 at Pusan National University Hospital. All included patients were pre-operatively chemo- and radiotherapy naïve. Also, all of them received adjuvant treatment. All patients were treated with 4 cycles of anthracycline and cyclophosphamide (AC) and 1 year of trastuzumab as a standard treatment. Paclitaxel or docetaxel was added between 4 cycles of AC and 1 year of trastuzumab based on the risk stratification encompassing the size of the tumour, lymph node metastasis, Ki-67 proliferation index, histologic grade, age, family history of breast cancer, and general condition. Among them, 14 patients discontinued trastuzumab during treatment, because 4 patients died, 11 patients had recurrence or metastasis and 3 developed cardiac toxicity. Formalin-fixed, paraffin-embedded tissue specimens were available for all patients. Clinicopathological information was collected by reviewing each patient’s electronic medical records and pathology reports. Among these, 39 fresh tissue samples were provided by the Pusan National University Hospital, a member of the National Biobank of Korea, supported by the Ministry of Health, Welfare, and Family Affairs. All samples from the National Biobank of Korea were obtained with informed consent under institutional review board-approved protocols.

### Histological evaluation

Histological evaluation of full face haematoxylin and eosin (H&E) slides was performed. Various histopathological factors, including histological grade, nuclear grade, necrosis, lymphovascular invasion, lymph node metastasis, TIL level, TLSs around the DCIS and invasive component were evaluated. Histological and nuclear grades were determined by the modified Bloom-Richardson classification. TILs level was assessed based on the recommendation by the International TILs Working Group^20^. TILs level was evaluated by visual estimation. In brief, TILs level was calculated as the percentage of stroma within the invasive area covered by mononuclear cells over total intratumoural stromal area. A full evaluation of average TILs in the tumour was performed without focusing solely on hotspots. The TILs level was evaluated as a percentage in 10% increments. If the TIL level was less than 10%, a 1% or 5% criteria was used^18^. The ducts affected by DCIS that had TLSs surrounding themselves of the total ducts affected by DCIS were assessed, and the amount of TLSs as a percentage of the total circumference of the invasive front was evaluated.

For statistical analysis, patients were subdivided into three categories (≤10%, 20–60%, and >60%) or two categories (≤60% and >60%) according to the TIL level as appropriate. TLSs level was divided into two subgroups (low and high) based on the mean value.

### Construction of tissue microarray blocks and immunohistochemical evaluation

All available H&E stained slides were reviewed to define a representative tumour area. A tissue arraying instrument was used to array each sample with a tissue core of 2 mm in diameter. To reduce tissue loss and the effects of tumour heterogeneity, all samples were arrayed in duplicate.

At the time of diagnosis, immunohistochemical staining of the oestrogen receptor (ER), progesterone receptor (PR), and HER2 was performed, and silver *in situ* hybridization (SISH) for HER2 was performed in cases of equivocal immunostaining with a score of 2. An automated immunohistochemical staining device (Benchmark XT; Ventana Medial System, Tuscon, AZ, USA) was used for immunohistochemical analysis. The EZ prep was used for deparaffinization with an auto-staining device on 3 µm paraffin sections. For antigen retrieval, cell conditioner 1 (CC1; pH 8.4 buffer) or cell conditioner 2 (CC2; pH 6.0 buffer) was used, and optimally formulated antibodies targeting ER (SP1, Ventana Medical Systems), PR (1E2, Ventana Medical Systems), and HER-2/neu (4B5, Ventana Medical Systems) were used. An anti-PD-L1 antibody (1:200, E1L3N, Cell Signaling Technology, Canvers MA, USA) was applied to the tissue microarray sections. A HER2/CEP17 chromosome dual-probe (INFROM HER2 Dual ISH DNA Probe Cocktail, Ventana Medical Systems) was used for SISH.

ER and PR positivity was defined as at least 1% of tumour nuclei showing positive staining. Tumours that were positive for ER or PR were classified as the HR-positive group. HER2 positivity was defined as an immunostaining score of 3, or by gene amplification identified by SISH. SISH was performed in cases of equivocal results, such as in the event of an immunohistochemical staining score of 2. The HER2 testing was interpreted according to American Society of Clinical Oncology/Cllege of American Pathologists Guideline 2007, which was used at the time of diagnosis^42^. Tumours were classified into two subtypes according to HR (ER and PR) expression: HR+/HER2+ and HR−/HER2+.

PD-L1 expression was evaluated separately for tumour cells and TILs. Membranous stains were assessed for PD-L1 positivity, using human placental tissue as a positive control. The intensity and the percentage of membranous positivity were considered collectively (Fig. 1). The immunoscore was calculated by multiplying the percentage of the positive cells by the staining intensity. The predominant staining intensity of 2 cores for each sample was used in cases of heterogeneous staining intensity. Total immunoscore was obtained by adding the tumour cells immunoscore and TILs immunoscore. For tumour cells positivity, the Allred score system was used^30, 43^. TILs expression levels were divided into two categories (low and high) based on the mean value of the PD-L1 immunoscore of the TILs. Additionally, tumours were classified into two categories (low and high) based on the mean value of the total PD-L1 immunoscore of tumour cells and TILs. For survival analysis, tumours were categorized into four groups according to the PD-L1 expression of the tumour cells and the TILs level, which is similar to the criteria of Teng’s classification, which are as follows: type I, positive for PD-L1 and high TIL level; type II, negative for PD-L1 and low TIL level; type III, positive for PD-L1 and low TIL level and type IV, negative for PD-L1 and high TIL level^21, 23^.

### Western blot analysis and quantitative real time-polymerase chain reaction (qRT-PCR)

Western blot analysis was performed on four HER2-positive breast cancer cases using fresh frozen tissue provided by the National Biobank Korea. Proteins from the fresh frozen tissue samples were loaded onto each well of the gel, separated by 12% sodium dodecyl sulphate polyacrylamide gel electrophoresis and transferred onto a membrane (Hybond P 0.45 PVDF, Amersham, Biosciences, Piscataway, NJ, USA). After blocking non-specific binding sites, the membrane was immunoblotted with the anti-PD-L1 antibody (1:1000, E1L3N, Cell Signaling Technology, Canvers MA, USA), followed by incubation with the secondary antibody conjugate. The bands were detected using an LAS-3000 Imaging System (Fujifilm, Tokyo, Japan).

Total mRNA was isolated from 39 cases of HER2 positive breast cancers provided by the National Biobank of Korea, using an RNeasy mini kit (Qiagen GmbH, Hilden, Germany) according to the manufacturer’s instructions. Extracted mRNA was reverse transcribed into cDNA using High-Capacity cDNA Reverse Transcription Kits (Applied Biosystems, Foster City, CA, USA) and amplified using TaqMan^®^ universal PCR Master mix (Applied Biosystems Inc., Foster City, CA, USA) and TaqMan^®^ Gene Expression Assays (Applied Biosystems Inc., Foster City, CA, USA). An ABI 7500 Real-Time PCR System (Applied Biosystems Inc., Foster City, CA, USA) was used for qRT-PCR. Each assay was performed in duplicate. The comparative CT method (ΔΔCT method) was used to determine the relative expression levels of PD-L1 mRNA. Relative expression levels of PD-L1 mRNA were normalized to those of beta-actin, and fresh human placental tissue was used for calibration. Expression levels of PD-L1 mRNA were subdivided into two categories (low and high) according the mean fold difference value.

### Statistical analysis

All statistical analyses were carried out using SPSS statistical software program (version11; IBM, Armonk, NY, USA). The t-test, Chi-square test, Fisher’s exact test, linear-by-linear association tests, Spearman rank correlation test, Kaplan-Meier survival analyses, log-rank test and Cox proportional hazards regression model were used as appropriate. For multivariate analysis, a forward stepwise selection method was used with covariates showing *p*-value of less than 0.10 in the univariate analyses. The likelyhood ratio test was done to ensure the proportional hazard assumptions in the Cox regression model. Differences with *p*-values of less than 0.05 were considered statistically significant.

### Ethical Approval and Informed Consent

Exemption from informed consent after de-identification of information was approved by the Institutional Review Board of Pusan National University Hospital.

## Electronic supplementary material

Supplementary Information
