# Supplementary material for: Programmed death-ligand 1 (PD-L1) expression in tumour cell and tumour infiltrating lymphocytes of HER2-positive breast cancer and its prognostic value
Source: Sci Rep. 2017 Sep 15;7:11671. doi: 10.1038/s41598-017-11905-7 (PMC5601941; doi:10.1038/s41598-017-11905-7)
Supplement: Supplementary file 1 — Supplementary Information [file 41598_2017_11905_MOESM1_ESM.pdf]

Programmed death-ligand 1 (PD-L1) expression in tumour cell and tumour infiltrating lymphocytes of HER2-positive breast cancer and its prognostic value

Ahrong Kim, MD, PhD[1], So Jeong Lee, MD[1], Young-Keum Kim, MD[1], Won-Young Park, MD[1], Do Youn Park, MD, PhD[1], Jee Yeon Kim, MD, PhD[2], Chang Hun Lee, MD, PhD[1], Gyungyub Gong, MD, PhD[3], Gi Young Huh, MD, PhD[1], Kyung Un Choi, MD, PhD[1]*

Author Affiliations:

[1]Department of Pathology, BioMedical Research Institute, Pusan National University Hospital, Pusan, Korea

[2]Department of Pathology, Pusan National University Yangsan Hospital, Yangsan, Korea

[3]Department of Pathology, Asan Medical Center, University of Ulsan College of Medicine, Seoul, Korea

Supplementary Table 1. Basic data for 167 HER2-positive breast cancers

Characteristic		Number (%)*
Age, mean ± SD**		52.59 ±10.126 years
Size, mean ± SD**		3.213±1.773 cm
Side		
	Left	93 cases (55.7)
	Right	74 cases (44.3)
Histologic grade		
	Grade 1, 2	62 (37.1)
	Grade 3	105 (62.9)
Nuclear grade		
	Grade 1, 2	29 (17.4)
	Grade 3	138 (82.6)
Necrosis		
	Absent	57 (34.1)
	Present	110 (65.9)
Lymphovascular invasion		
	Absent	90 (53.9)
	Present	77 (46.1)
Lymph node metastasis		
	Absent	78 (46.7)
	Present	89 (53.3)
Stage		
	Early (stage I, II)	126 (75.4)
	Advanced (stage III, IV)	41 (24.6)
HR expression		
	Positive	89 (53.3)
	Negative	78 (46.7)
TILs level		
	≤10%	61 (36.5)
	20-60%	74 (44.3)
	>60%	32 (19.2)
TLSs around DCIS		
	Low	102 (61.1)
	High	65 (38.9)
TLSs around invasive component		
	Low	109 (65.3)
	High	58 (34.7)

*except for age and size **SD, standard deviation

Supplementary Table 2. PD-L1 expression and TILs level in total 167 HER2-positive breast cancers

Characteristic		Number (%)
PD-L1 expression in tumour cells		
	Immunoscore, mean±SD*	7.03±20.853
	Negative by Allred system	86 (51.5)
	Positive by Allred system	81 (48.5)
Immunoscore of PD-L1 in TILs		
	Immunoscore, mean±SD	15.38±23.135
	Low	116 (69.5)
	High	51 (30.5)
Total immunoscore of PD-L1 in both tumour cells and TILs		
	Immunoscore, mean±SD	22.41±35.921
	Low	114 (68.3)
	High	53 (31.7)
Tumour type according to Teng's classification		
	Type I	28 (16.8)
	Type II	82 (49.1)
	Type III	53 (31.7)
	Type IV	4 (2.4)

*SD, standard deviation

Supplementary Table 3. Correlation of PD-L1 expression in tumour cells and TILs*

	PD-L1 expression in tumour cells by Allred scoring system		p-value
	Negative (Number (%))	Positive (Number (%))	
PD-L1 expression level in TILs			<0.001
Low	78 (90.7)	38 (46.9)	
High	8 (9.3)	43 (53.1)	

*The Pearson's Chi-square test was used.

Supplementary Table 4. Disease-free survival based on various clinicopathologic parameters in HR-/HER2+ breast cancers*

	Univariate analysis			Multivariate analysis		
	HR**	95% CI***	*p*-value	HR	95% CI	*p*-value
Stage (I,II vs III, IV)	15.453	3.370-70.849	*P*<0.001	9.760	2.152-44.263	*P*=0.013
T stage (1,2 vs 3,4)	6.091	1.955-18.979	*P*=0.002	1.626	0.566-4.671	*P*=0.449
Lymph node metastasis	63.050	0.605-6570.270	*P*=0.080			
Lymphovascular invasion	6.774	1.482-30.956	*P*=0.014	1.692	0.395-7.245	*P*=0.552
TILs (0-60% vs >60%)	0.027	0.000-4.536	*P*=0.167			
PD-L1	0.277	0.075-1.022	*P*=0.054	0.336	0.105-1.076	*P*=0.123

*Cox proportional hazard regression model was used.

**HR, hazard ratio

***CI, confidence interval

Supplementary Figure 1. Western blot analysis of 4 fresh tissue samples with HER2-positive breast cancers

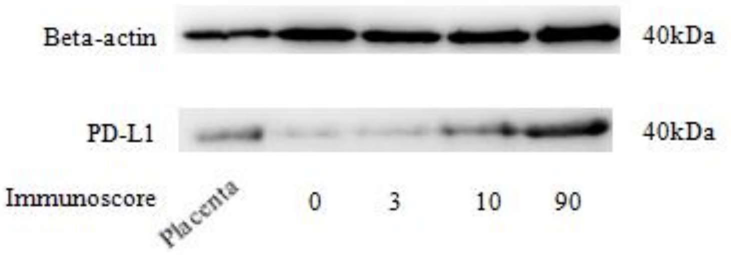

Supplementary Figure 2. Overall survival and disease-free survival in 167 HER2-positive breast cancers (A) Overall survival according to PD-L1 expression in tumour cells (B) Disease-free survival according to PD-L1 expression in tumour cells (C) Overall survival according to level of PD-L1 immunoscore in TILs (D) Disease-free survival according to level of PD-L1 immunoscore in TILs (E) Overall survival according to level of total PD-L1 immunoscore (F) Disease-free survival according to level of total PD-L1 immunoscore (G) Overall survival according to tumour types (H) Disease-free survival according to tumour types

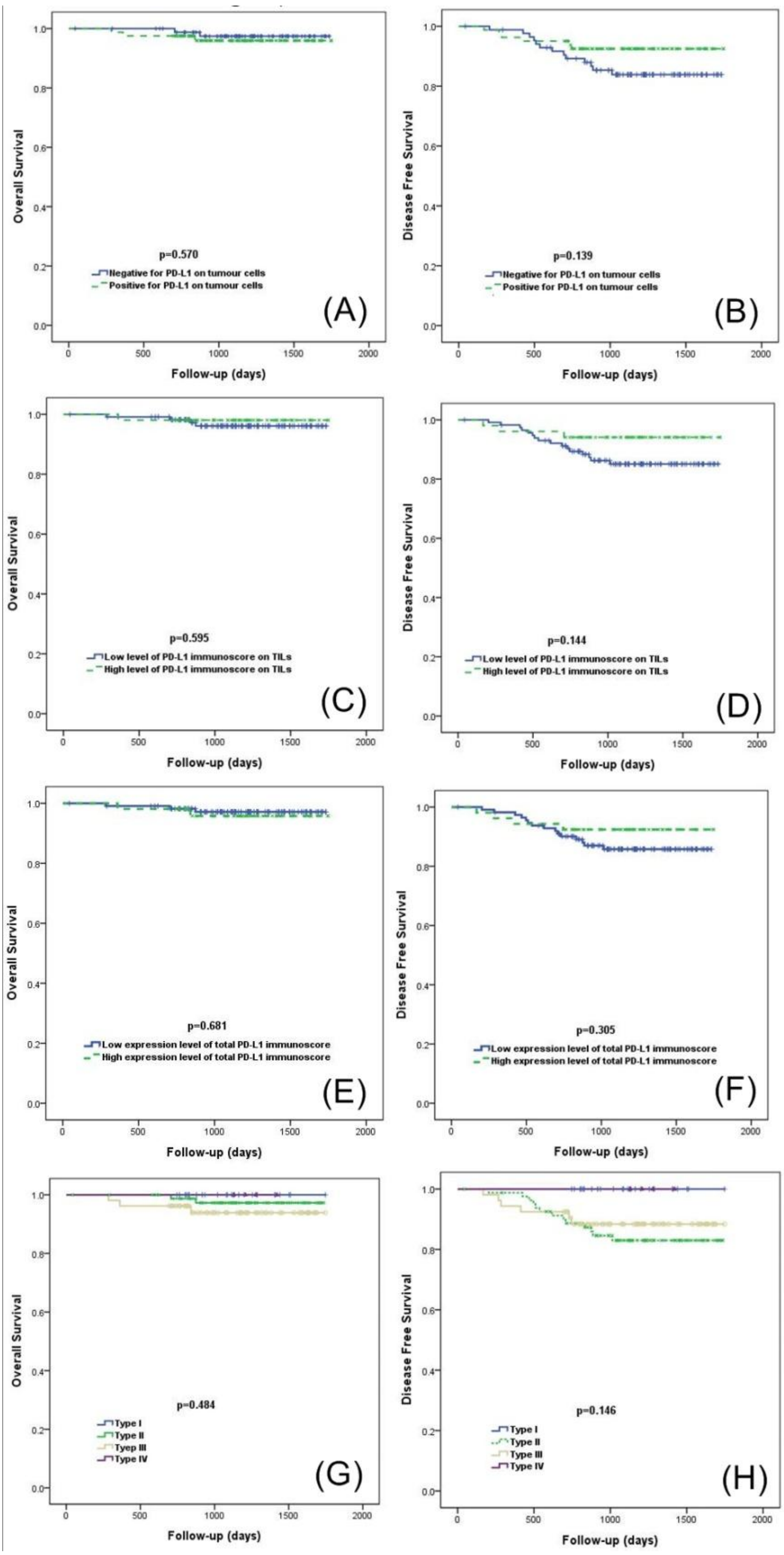
p=0.570
Negative for PD-L1 on tumour cells
Positive for PD-L1 on tumour cells
(A)
p=0.139
Negative for PD-L1 on tumour cells
Positive for PD-L1 on tumour cells
(B)
p=0.595
Low level of PD-L1 immunoscore on TILs
High level of PD-L1 immunoscore on TILs
(C)
p=0.144
Low level of PD-L1 immunoscore on TILs
High level of PD-L1 immunoscore on TILs
(D)
p=0.681
Low expression level of total PD-L1 immunoscore
High expression level of total PD-L1 immunoscore
(E)
p=0.305
Low expression level of total PD-L1 immunoscore
High expression level of total PD-L1 immunoscore
(F)
p=0.484
Type I
Type II
Tyep III
Type IV
(G)
p=0.146
Type I
Type II
Type III
Type IV
(H)
Overall Survival
Disease Free Survival
Follow-up (days)

Supplementary Figure 3. Overall survival and disease-free survival in HR+/HER2+ breast cancers (A) Overall survival according to PD-L1 expression in tumour cells (B) Disease-free survival according to PD-L1 expression in tumour cells (C) Overall survival according to level of PD-L1 immunoscore in TILs (D) Disease-free survival according to level of PD-L1 immunoscore in TILs (E) Overall survival according to level of total PD-L1 immunoscore (F) Disease-free survival according to level of total PD-L1 immunoscore (G) Overall survival according to tumour types (H) Disease-free survival according to tumour types

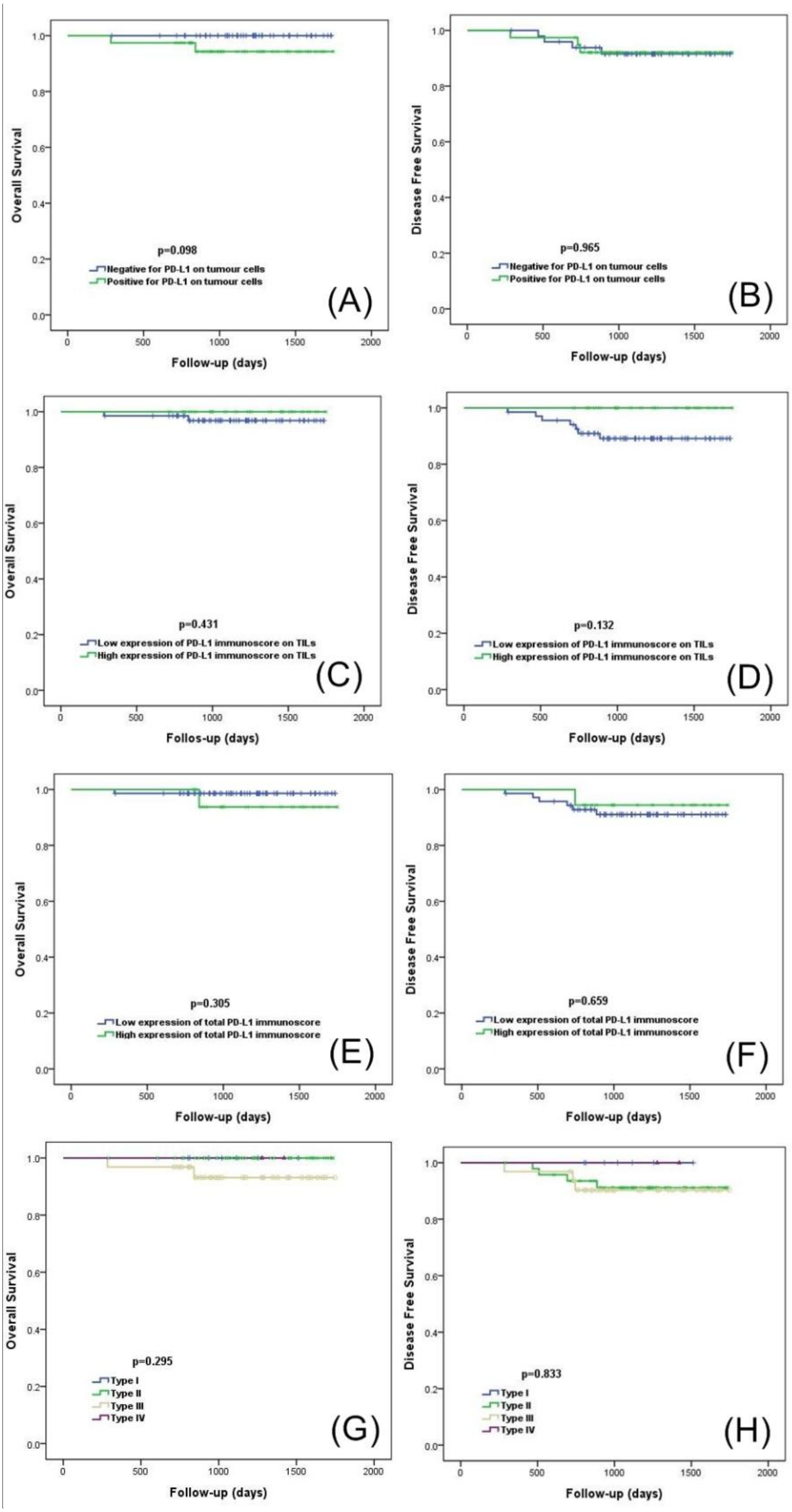

Overall Survival
p=0.098
Negative for PD-L1 on tumour cells
Positive for PD-L1 on tumour cells
(A)
Follow-up (days)
Disease Free Survival
p=0.965
Negative for PD-L1 on tumour cells
Positive for PD-L1 on tumour cells
(B)
Follow-up (days)
Overall Survival
p=0.431
Low expression of PD-L1 immunoscore on TILs
High expression of PD-L1 immunoscore on TILs
(C)
Follos-up (days)
Disease Free Survival
p=0.132
Low expression of PD-L1 immunoscore on TILs
High expression of PD-L1 immunoscore on TILs
(D)
Follow-up (days)
Overall Survival
p=0.305
Low expression of total PD-L1 immunoscore
High expression of total PD-L1 immunoscore
(E)
Follow-up (days)
Disease Free Survival
p=0.659
Low expression of total PD-L1 immunoscore
High expression of total PD-L1 immunoscore
(F)
Follow-up (days)
Overall Survival
p=0.295
Type I
Type II
Type III
Type IV
(G)
Follow-up (days)
Disease Free Survival
p=0.833
Type I
Type II
Type III
Type IV
(H)
Follow-up (days)
